# Integrative RNA-Seq and ATAC-Seq Analysis Reveals the Migration-Associated Genes Involved in Antitumor Effects of Herbal Medicine Feiyanning on Lung Cancer Cells

**DOI:** 10.3389/fgene.2021.799099

**Published:** 2021-12-21

**Authors:** Chenyang Wang, Pengxiao Li, Yonglin Peng, Ruiqi Liu, Xiaoting Wu, Sheng Tan, Ming Zhang, Xiaodong Zhao

**Affiliations:** ^1^ Shanghai Center for Systems Biomedicine, Key Laboratory of Systems Biomedicine (Ministry of Education), Shanghai Jiao Tong University, Shanghai, China; ^2^ Department of Integrated Traditional Chinese and Western Medicine, Shanghai Chest Hospital, Shanghai Jiao Tong University, Shanghai, China

**Keywords:** traditional Chinese medicine, Feiyanning, non-small cell lung cancer, Migration, RNA-seq, ATAC-seq

## Abstract

Lung cancer is one of the leading causes of cancer-associated death in the world. It is of great importance to explore new therapeutic targets. Traditional Chinese medicine formula Feiyanning has been clinically administered in China for more than a decade and raised attention due to its anticancer effect in lung cancer. However, the underlying molecular mechanisms remain to be elucidated. In the present study, we carried out cellular and molecular assays to examine the antitumor activities and understand the mechanism of the Feiyanning formula in lung cancer cells. The cellular viability of Feiyanning-treated lung cancer cells was evaluated by Cell Counting Kit-8. The effect of the Feiyanning formula on cellular migration and invasion of lung cancer cells was examined by wound healing and transwell assays. Transcriptome and chromatin accessibility analysis by RNA-seq and ATAC-seq was performed to investigate the underlying molecular mechanisms. Our results revealed that the Feiyanning formula inhibited the cellular activities of proliferation, migration, and invasion in non-small cell lung cancer cell lines A549, H1975, and 95D. Furthermore, we observed that the transcriptional activity of the migration-associated genes was downregulated upon Feiyanning formula treatment in non-small cell lung cancer cells. The chromatin accessibility of the Feiyanning-treated lung cancer genome tended to decrease, and the regulation of the cellular component movement biological process and PI3K-AKT pathway were enriched among these altered genomic regions. Taken together, the present study suggested that Feiyanning formula exerted the antitumor effects by modulating the expression and chromatin accessibility levels of migration-associated genes.

## Introduction

Lung cancer is a malignancy with high morbidity and mortality around the world (Siegel et al., 2018; Cao et al., 2020). Similar to other types of cancer, lung cancer possesses many characteristics, such as rapid invasion ability (Popper, 2016). To make matters worse, the majority of lung cancer patients are already at the late stage when the initial diagnosis is performed (Gridelli et al., 2015). Lung cancer patients are routinely treated with surgery (Hoy et al., 2019), radiation therapy (Aokage et al., 2017; Gensheimer and Loo, 2017; Fitzgerald and Simone, 2020), chemotherapy (Nagasaka and Gadgeel., 2018), immunotherapy (Steven et al., 2016; Doroshow et al., 2019), targeted therapy (Hirsch et al., 2017; Duma et al., 2019), or a combination of these treatments. However, these treatments have both advantages and disadvantages. For example, radiotherapy has an effect on cancer cells; however, it simultaneously causes damage to nonmalignant cells and function of the body. Although targeted therapy does not cause damage to normal cells, this therapy is effective only for patients with mutated genes, and it inevitably induces drug tolerance after treatment for a period of time (Molina et al., 2008; Kalia, 2015; Arbour and Riely., 2019; Rapoport and Anderson., 2019). Therefore, it is important to look for alternative treatments.

During the past decades, more and more natural remedies have been used in cancer therapy (Newman and Cragg., 2014). They have few side effects and can improve life quality (Xu et al., 2014). Feiyanning (FYN), a Chinese herbal formula consisting of 11 herbs, has been clinically administered for more than a decade with benefit of prolonging patients’ survival (Gong et al., 2018). Very recently, it has been reported that the FYN formula can induce apoptosis of lung cancer cells through activation of the mitochondrial pathway (Zhu et al., 2021). However, the antitumor mechanism of the FYN formula has not been completely elucidated. In recent years, we have performed a mechanistic characterization of the herbal formula. For example, we reported that Jinfukang, a Traditional Chinese medicine against lung cancer, could induce cellular apoptosis (Lu et al., 2018). Moreover, we observed that Jinfukang could improve cytotoxicity when lung cancer cells are treated with the combination of Jinfukang and cisplatin, the first-line chemotherapy drug for lung cancer (Lu et al., 2016).

In this study, we carried out cellular assays to examine the effect of the FYN formula on non-small cell lung cancer cell (NSCLC) lines and performed deep-sequencing analysis to understand the underlying mechanism. We observed that the FYN formula suppressed the cellular activities of proliferation, migration, and invasion in lung cancer cells and exerted the antitumor effects *via* the transcriptional regulation of cell migration- and death-related genes. This study suggested that the FYN formula could be a potential adjunct or even therapeutic choice for lung cancer patients and provide insight into understanding the antitumor mechanism of FYN formula.

## Materials and methods

### Preparation of FYN formula

The FYN formula consists of *Astragalus membranaceus* (30 g), *Polygonatum sibiricum* (30 g), *Cornus officinalis* (15 g), *Paris polyphylla* (9 g), *Atractylodes macrocephala* (9 g), *Polistes olivaceus* (9 g), *Salvia chinensis* (30 g), *Corium bufonis* (6 g), *Ganoderma lucidum* (15 g), *Pseudobulbus cremastrae seu pleiones* (15 g), and *Epimedii folium* (15 g). The herb medicine was obtained from the pharmacy of Shanghai Chest Hospital, Shanghai Jiao Tong University (Shanghai, China). The following components were mixed as follows: distilled deionized water was added to the herb mixture (solid–liquid ratio: 1:5) and soaked for 1 h. The mixture was kept warm for about 1 h and filtered with six layers of gauze. Then, the filtrate was immediately kept at −70°C. Finally, the lyophilized powder of the FYN formula was prepared by freeze-drying at −50°C for 72 h. The prepared FYN formula frozen powder was divided into 15–20 g/tube for the subsequent cellular assays. Mass spectrographic fingerprints for the FYN formula extract were examined for quality check. The lyophilized powder was diluted to various concentrations for the subsequent assays in the present study.

### Cell culture

NSCLC cell lines A549, 95D, and H1975 were provided by the Cell Bank of Chinese Academy of Sciences (Shanghai, China) and maintained with RPMI 1640 medium (Invitrogen, Carlsbad, CA, USA). The culture medium contained 1% penicillin/streptomycin (Invitrogen, Carlsbad, USA) and 10% fetal bovine serum (FBS) (Invitrogen, Carlsbad, USA). Lung cancer cells were cultured in an incubator at 37°C with 5% CO2. *Mycoplasma* contamination was monitored through PCR to make sure that all cell lines in this study were mycoplasma-free.

### Cell viability assay

The cell viability was examined by the Cell Counting Kit-8 (CCK-8, Sangon, Shanghai, China). Briefly, cells were seeded at a density of 3 × 10^3^ cells/well and cultured in 96-well plates overnight. Lung cancer cells were treated with different concentrations of FYN formula (0, 0.25, 0.5, 1, 2, and 3 mg/ml) for 24, 48, and 72 h, respectively. After treatment, 10 μl CCK-8 solution was added into each well and incubated for 3 h. The measurement of absorbance was carried out at 450 nm with a spectrophotometric plate reader (Omega Bio-Tek, Norcross, GA, USA). Three independent experiments were carried out with five replicates in each group.

### Wound healing assay

Lung cancer cells were seeded at the density of 1 × 10^6^ cells per well into 6-well plates and incubated overnight. When lung cancer cells reached 90% confluence, they were treated with the FYN formula for 24 h. In a central position of each well, a horizontal scratch was made with a 10-μl tip. The wound closure was then observed, and the images were captured at 0 and 24 h by microscope (Nikon, Tokyo, Japan). The scratch healing degree was used to determine the migration ability. Three independent experiments were carried out.

### Transwell assay

In the migration assay, 2 × 10^4^ lung cancer cells were seeded into the upper chamber (Corning, NY, USA). For the invasion assay, we diluted Matrigel and added it to the 24-well invasion chambers and maintained overnight at 37°C. A total of 4 × 10^4^ cells were seeded into the upper well of the chamber. In order to stimulate cell migration, medium with 15% FBS was added into the lower chamber. After incubation for 24 h, lung cancer cells were fixed for 30 min and stained with 0.1% crystal violet solution for 20 min at room temperature. The non-invaded cells at the top were removed, while the cells at the bottom were photographed in three independents 20 × fields for each well. Three independent experiments were conducted.

### Preparation of strand-specific RNA-Seq library and deep sequencing

Total RNA was extracted using TRIzol (Thermo Fisher Scientific, Waltham, MA, USA), and mRNA was purified using the NEBNext Poly(A) mRNA Magnetic Isolation Module Kit (NEB, New England, Ipswich, MA, USA). A strand-specific RNA-seq library was generated with NEBNext Ultra Directional RNA Library Prep Kit (NEB, New England, USA). Briefly, mRNA was fragmented into small fragments and reversely transcribed into cDNA. Then, second-strand cDNA was generated. Next, the double-strand DNA fragments were purified with AMPure beads (Beckman Coulter, Brea, CA, USA) and ligated with adapters. The resulting ligation products were amplified and sequenced with HiSeq X (Illumina, San Diego, CA, USA). The raw sequencing data could be obtained in the EMBL database (http://www.ebi.ac.uk/arrayexpress/) under accession number E-MTAB-10821. The sequencing data of the control we generated in our previous study are available in the EMBL database (http://www.ebi.ac.uk/arrayexpress/) under accession number E-MTAB-7237 (Yang et al., 2019).

### Preparation of ATAC-Seq library and deep sequencing

A total of 50,000 cells were resuspended for nucleus isolation. Chromatin was fragmented using Tn5 transposase (Vazyme, Nanjing, China) and followed by amplification (9 cycles). Lastly, the resulting libraries were purified with the Xygen purification kit (Xygen, USA). The distribution of library fragments was examined by the Agilent 2100 bioanalyzer. Libraries were sequenced on HiSeq X. The raw sequencing data could be obtained in the EMBL database (http://www.ebi.ac.uk/arrayexpress/) under accession number E-MTAB-11023.

### Bioinformatics analysis

The raw sequencing reads generated in this study were mapped to the human genome (hg19) using TopHat v2.1.1 (Trapnell et al., 2012). For RNA-seq, differentially expressed gene analysis was carried out by comparing genes in A549 and those in A549 cells treated with FYN using cuffdiff v2.2.1. Cufflinks v2.2.1 (Trapnell et al., 2012) was used to quantify their expression. The gene expression levels were measured by fragments per kilobase of transcript per million reads. Gene ontology (GO) analysis was performed using Gene Ontology Resource (http://geneontology.org/). DAVID was used for KEGG pathway analysis (https://david.ncifcrf.gov/tools.jsp). For ATAC-seq, peaks of each sample were called with MACS2. A heatmap was generated using deepTools (v3.3.0) (Ramírez et al., 2016). To visualize the ATAC-seq signals using Integrative Genomics Viewer (IGV, v2.5.3), the bam files were transformed into bigwig (bw) files with deepTools (Robinson et al., 2011). We applied HOMER (v4.4.1) to perform differential chromatin accessibility (DA) analysis with the getDifferentialPeaks algorithm (Heinz et al., 2010). Both RNA-seq and ATAC-seq data sets were analyzed with default parameters, and the *p* values were corrected by conventional FDR. Notably, the promoter regions were defined as—1,000 bp/+ 100 bp of nearest transcription start sites. If a peak falls in the 5 ′UTR region, it is preferentially assigned to the 5′ UTR region rather than the promoter region.

### Statistical analysis

Data were exhibited as the mean ± standard deviation (SD). The differences between each group were examined with GraphPad Prism 8.0 software. We considered the results with *p < 0.05, **p < 0.01, and ***p < 0.001 to be significantly different.

## Results

### FYN suppresses growth of NSCLC cells

**FIGURE 1:**
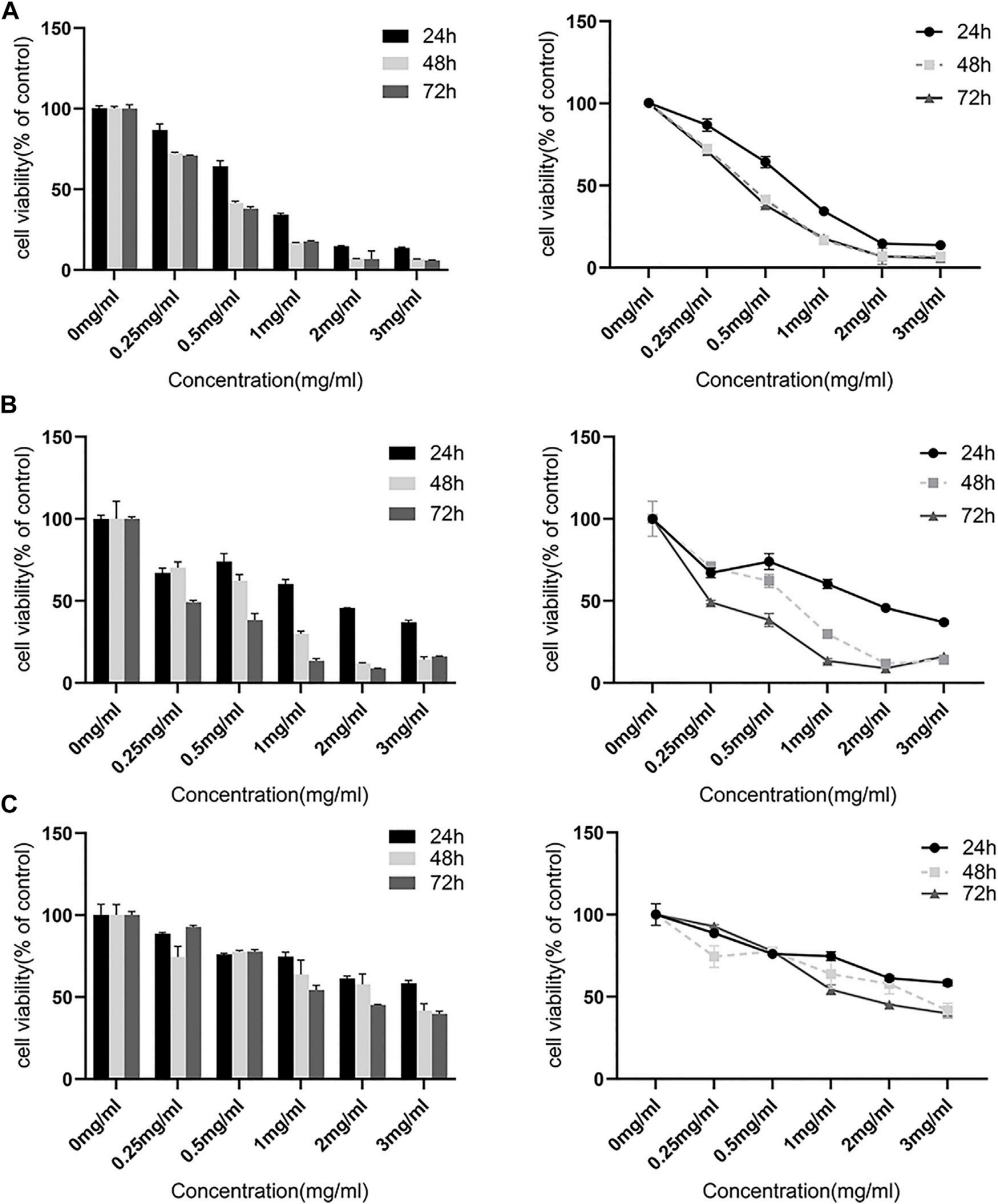
FYN suppresses the cellular growth of lung cancer cells. The CCK-8 kit was used to detect cell viability after 24, 48, and 72 h. Data were presented as the means ± SD from three independent experiments with five replicates per experiment. **(A)**: A549, **(B)**: H1975, **(C)**: 95D.

The extract of the FYN formula was prepared as reported previously (Zhu et al., 2021). CCK-8 assay was performed to examine the effect of the FYN formula on the viability of lung cancer cell lines A549, H1975, and 95D. As shown in Figures 1A, B, the FYN formula significantly inhibited the viability of A549 and H1975 in a dose-dependent manner after the treatment for 24, 48, and 72 h. In addition, the FYN formula also inhibited the viability of 95D (Figure 1C). However, we found that A549 and H1975 were more sensitive to the FYN-induced cell viability attenuation than 95D. The half inhibitory concentration (IC50) of the FYN formula in A549, H1975, and 95D was 0.73, 1.56, and 4.68 mg/ml, respectively.

### FYN inhibits NSCLC cellular migration and invasion

**FIGURE 2:**
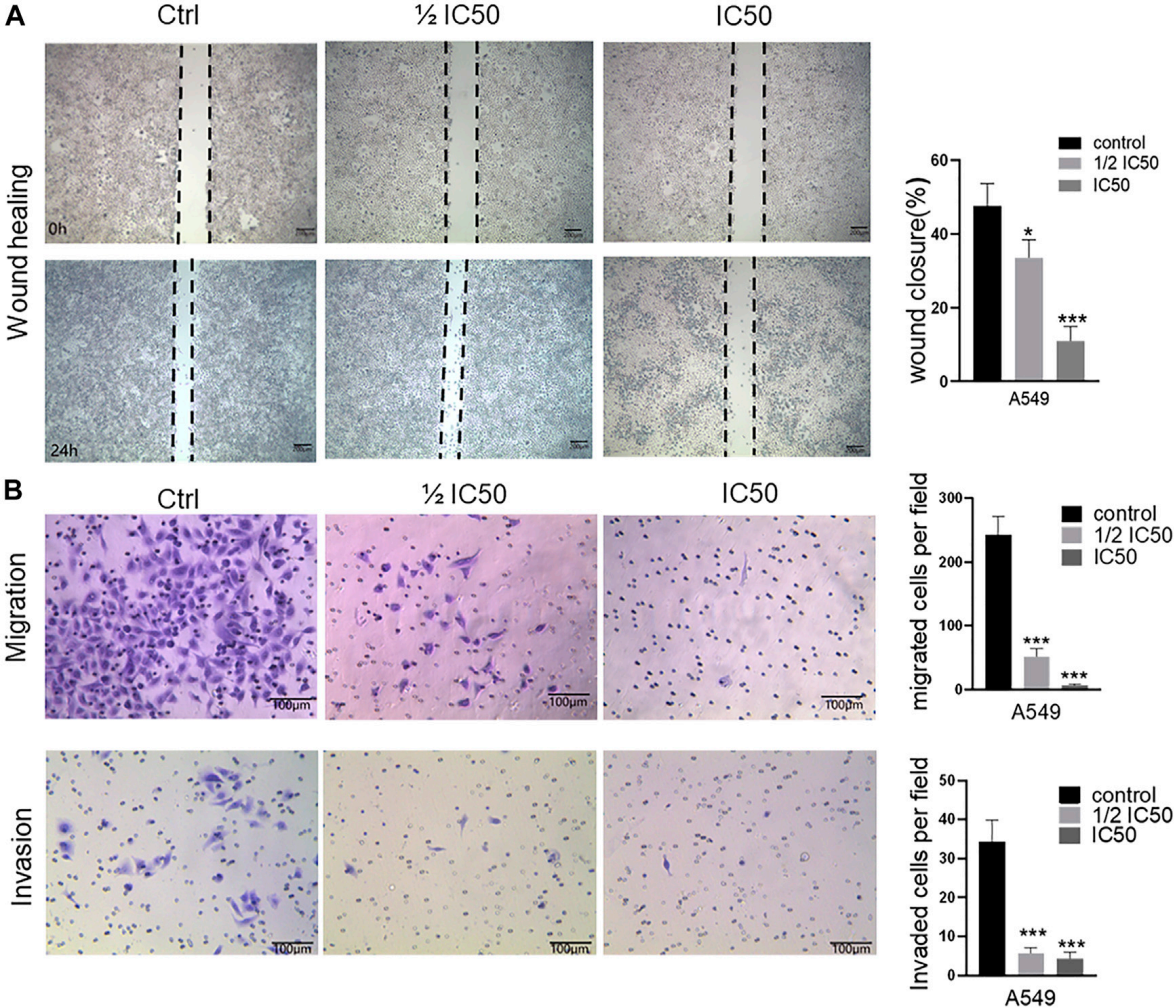
FYN suppresses the cellular migration and invasion of A549 cells. FYN-treated A549 were used for wound healing assay (10 × field) and invasion assay (20 × field). Data were presented as the means ± SD from three independent experiments. *, **, and *** indicate significant difference compared to the control group at *p* < 0.05, *p* < 0.01, and *p* < 0.001, respectively. **(A)**: Wound healing assay. **(B)**: Transwell assay.

To further explore the anticancer effect of the FYN formula, we performed wound healing assay to examine whether FYN affects the migratory activity of lung cancer cells. We observed that the cells without treatment (the control) rapidly migrated, but the migration area of FYN-treated cells was significantly decreased and this trend was more obvious when the FYN formula concentration was increasing. After a 24-h treatment with various concentrations of the FYN formula, the migration inhibition rates of A549 cells were 66.5 ± 5.0% (0.365 mg/ml, *p* < 0.05) and 89.1 ± 3.9% (0.73 mg/ml, *p* < 0.001), respectively (Figure 2A). For H1975 and 95D cells, the migration inhibition rates were 81.8 ± 10.3% (0.78 mg/ml, *p* < 0.01), 89.9 ± 3.8% (1.56 mg/ml, *p* < 0.001) and 80.8 ± 6.3% (2.34 mg/ml, *p* < 0.001), 84 ± 5.2% (4.68 mg/ml, *p* < 0.001), respectively (Supplementary Figures S1A, B). These results showed that the FYN formula could effectively decrease the migration of lung cancer cells examined.

We next performed transwell assay to further investigate the inhibitory effect of the FYN formula on the migration and invasion of A549, H1975, and 95D cells. We found that the numbers of A549, H1975, and 95D cells that migrated to the lower chamber declined in a concentration-dependent manner after a 24-h treatment (Figure 2B, Supplementary Figure S2). Furthermore, the density of the FYN-treated cells declined as well (Figure 2B, Supplementary Figure S2). These results further indicated that the FYN formula could obviously inhibit the migration ability of A549, H1975, and 95D cells.

### FYN-associated transcriptome analysis in treated A549 cells

**FIGURE 3:**
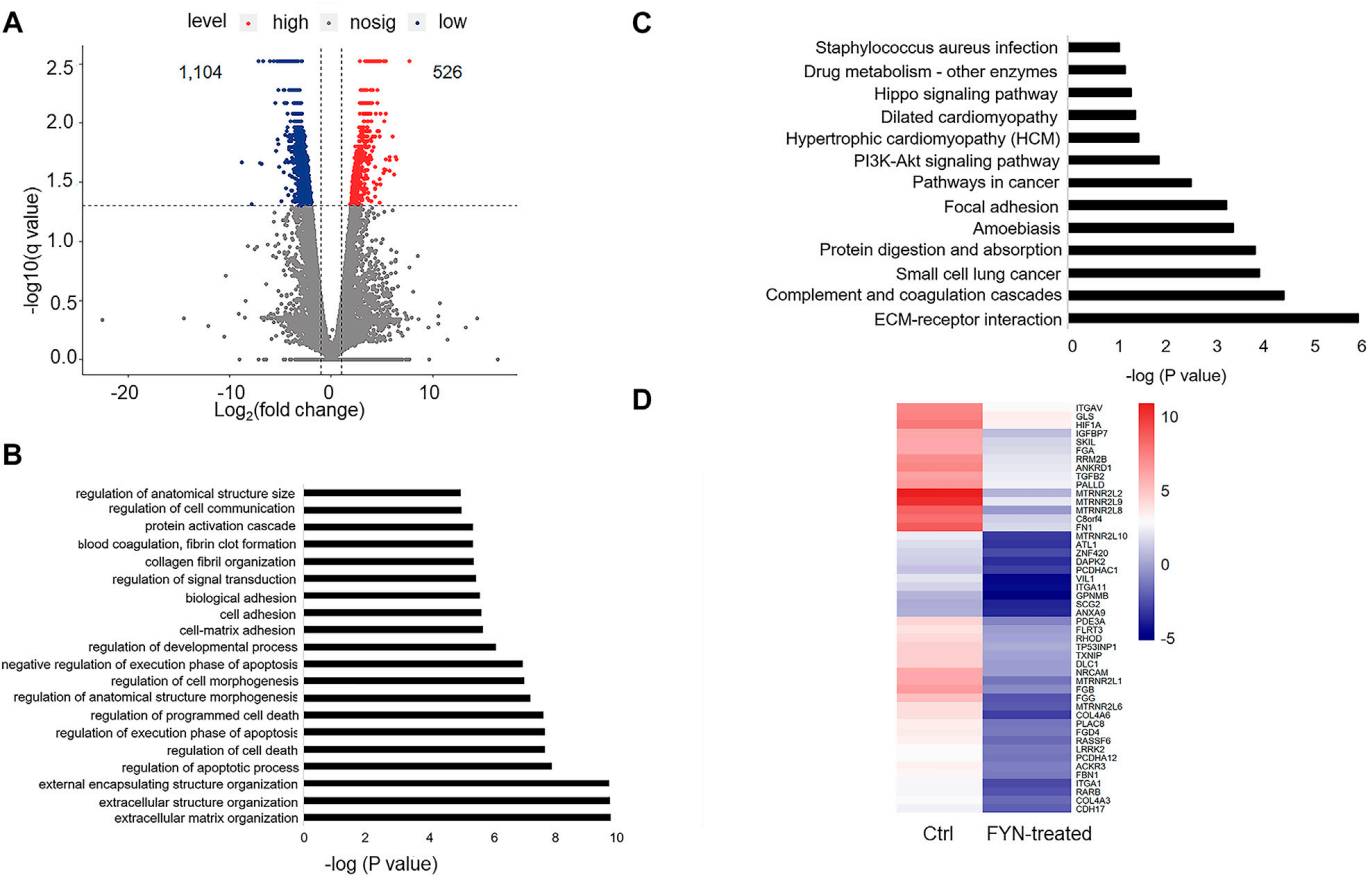
Characterization of the FYN-induced gene expression alteration in lung cancer cells. **(A)**: Scatter plot indicated the pattern of differential gene expression in FYN-treated A549 cells and the counterpart of A549 cells without FYN treatment, including both upregulated (red) and downregulated (blue) genes. **(B)**: GO analysis of the downregulated genes in FYN-treated A549. **(C)**: KEGG analysis of the downregulated genes in A549 treated with FYN. **(D)**: Heatmap of downregulated genes related to apoptosis and cell adhesion in A549 treated with FYN.

Since we observed the anti-proliferation and anti-migration activities of the FYN formula, we next aimed to investigate the underlying molecular mechanisms through RNA-seq analysis. A549 cells are most sensitive to FYN among the NSCLC cells examined; we then generated RNA-seq data of FYN-treated A549 cells and set the data of A549 without FYN treatment as the control. Compared with the control, we identified 1,630 differentially expressed genes (adjusted *p*-values ≤ 0.05, |log_2_fold change| ≥ 1) in FYN-treated A549; 526 genes were upregulated and 1,104 genes were downregulated (Figure 3A; Supplementary Table S1). GO analysis of obviously downregulated genes (log_2_ fold change < -4) (Supplementary Table S2) indicated that the GO terms of migration- and death-related biological processes were statistically enriched (Figure 3B). In addition, we carried out KEGG pathway analysis with the differentially expressed genes. As expected, we observed that the focal adhesion and PI3K-AKT pathways were enriched (Figure 3C). The transcriptional change in cell adhesion- and apoptosis-related genes was shown in Figure 3D. These results suggested that the FYN formula may exert anticancer effects through inhibition of cellular proliferation and migration.

### Identifying altered chromatin accessible regions in FYN-treated A549 cells

**FIGURE 4:**
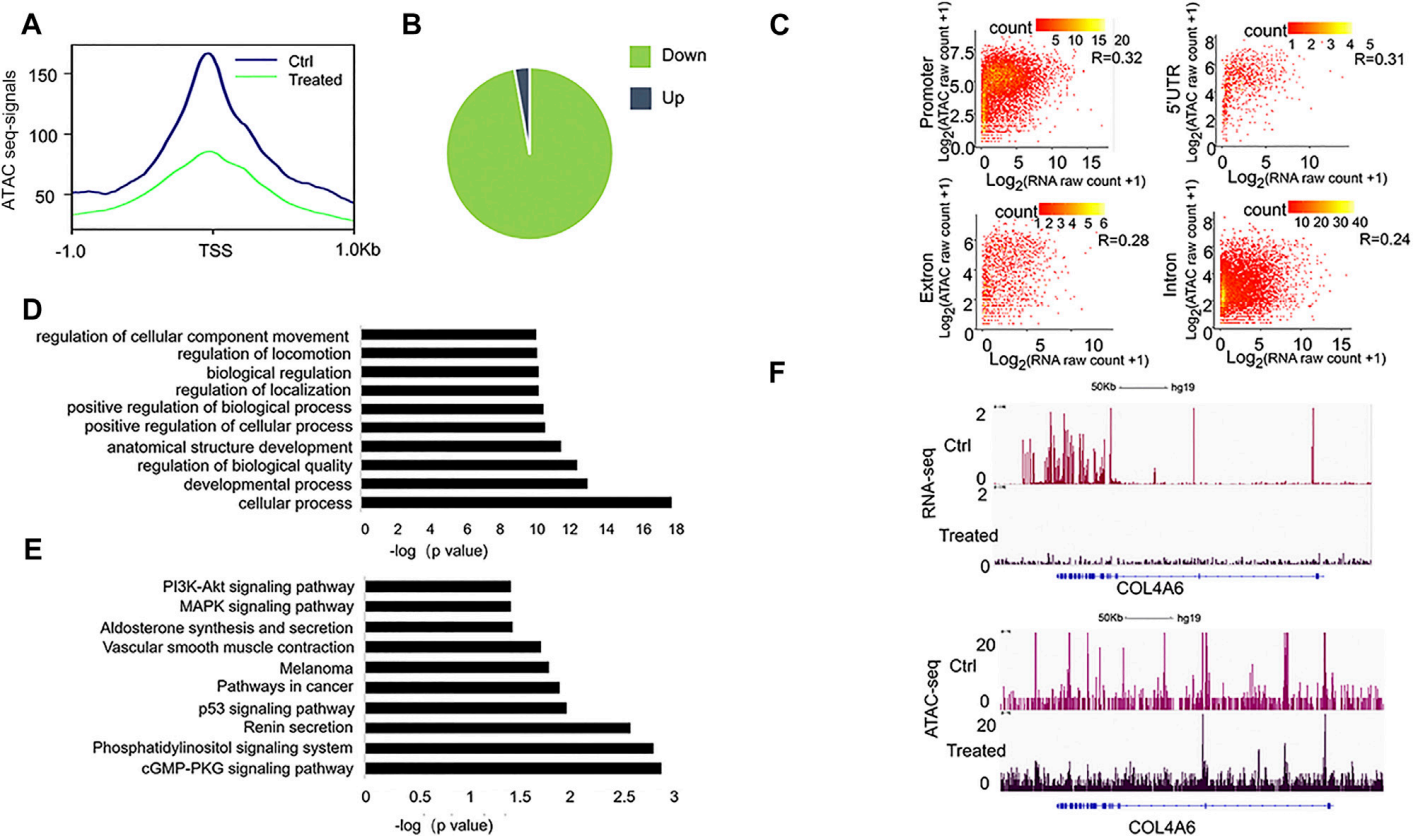
Characterization of genome-wide chromatin accessibility in FYN-treated A549 cells. **(A)**: Line plot shows the decreased chromatin accessibility in FYN-treated A549 cells around the TSSs of the nearest genes. **(B)**: The pie chart shows the chromatin accessibility levels after FYN treatment. **(C)**: Point density plots indicating the correlation between ATAC-seq total tags and RNA-seq expression levels at the promoter, 5′UTR, exon, and intron regions in FYN-treated A549. **(D)**: GO analysis of differentially accessible peaks. **(E)**: KEGG of differentially accessible peaks. **(F)**: IGV browser snapshots at the *COL4A6* locus showing a lower expression level and decreased chromatin accessibility.

It has been recognized that the open chromatin-accessible regions contain cis-regulatory elements and might modulate gene activity (Frank et al., 2015). We then performed chromatin accessibility profiling analysis in both A549 (the control) and FYN-treated A549 cells through ATAC-Seq. We identified 101,767, and 25,796 accessible regions in the control and FYN-treated A549, respectively. Specifically, we observed that the chromatin accessibility near transcription start sites (1 kb around transcription start sites) tended to decrease upon FYN treatment (Figure 4A, Supplementary Figure S3A). Compared with the control, the chromatin accessibility levels of 48,290 ATAC peaks were observed to be altered (|log_2_ fold change | ≥ 2) in FYN-treated A549 cells, among which 97% were downregulated and 3% were upregulated (Figure 4B). Most of these altered accessibility regions were located in intronic and intergenic regions (Supplementary Figure S3B). While detecting the relationship between RNA expression levels and chromatin accessibility levels at each genomic region, we found the correlation at the gene promoter in FYN-treated A549 was relatively large (Figure 4C, Supplementary Figure S4).

Then, the obviously downregulated peaks (log_2_ fold change < −5) were annotated to the nearest gene (Supplementary Table S3) and we performed GO and KEGG analyses. We found that GO item regulation of cellular component movement (Figure 4D) and PI3K-AKT signaling pathway (Figure 4E) were enriched, which is consistent with the observation from RNA-seq analysis. For example, in FYN-treated A549 cells we found the decreased chromatin accessibility and lower expression level at the *COL4A6* (Figure 4F), a member of the PI3K-AKT signaling pathway that was reported to be involved in cell metastasis (Ma et al., 2020). These findings suggested that the migration-associated genes were involved in the antitumor effect of FYN formula.

## Discussion

There is mounting evidence that herbal medicines play increasingly important roles in cancer treatment, as adjunctive treatments or even therapeutic agents (Liu et al., 2012; Jiang et al., 2016). The FYN formula is composed of 11 herbs, which has been found to have anticancer efficacy in lung cancer (Xu et al., 2011). In this study, we observed that the FYN formula exerted antitumor effects by inhibiting the cellular proliferation, migration, and invasion of A549, H1975, and 95D cells. Furthermore, we found that FYN treatment suppressed the expression of migration- and focal adhesion-associated genes and altered chromatin accessibility. In particular, among the genes with suppressed expression levels and decreased chromatin accessible regions, we found that the PI3K-AKT pathway was enriched (Figures 3C, 4E). The PI3K-AKT pathway has been reported to be activated in NSCLC, and some small compound inhibitors of PI3K and AKT were developed for clinical trials in NSCLC patients (Tan, 2020). In our study, we found that the FYN formula also inhibited the PI3K-AKT pathway, which plays a similar role as those small compound inhibitors do. Collectively, these findings may provide new evidence to support the antitumor potential of the FYN formula as an adjunctive treatment for NSCLC.

We were aware of the limitations of this research. Firstly, the biological consequence of FYN-induced expression alteration of cell growth- and migration-related genes needs further investigation. Secondly, in this study we carried out cellular assays and differential gene expression analysis. It would be more informative to validate the present findings in the mouse xenograft of human lung cancer.

## Data Availability

The datasets presented in this study can be found in online repositories. The names of the repository/repositories and accession number(s) can be found in the article/Supplementary Material.
