# Supplementary material for: Integrative RNA-Seq and ATAC-Seq Analysis Reveals the Migration-Associated Genes Involved in Antitumor Effects of Herbal Medicine Feiyanning on Lung Cancer Cells
Source: Front Genet. 2021 Dec 21;12:799099. doi: 10.3389/fgene.2021.799099 (PMC8724546; doi:10.3389/fgene.2021.799099)
Supplement: Supplementary file 3 [file DataSheet1.PDF]

Supplementary Material

Supplementary Figures

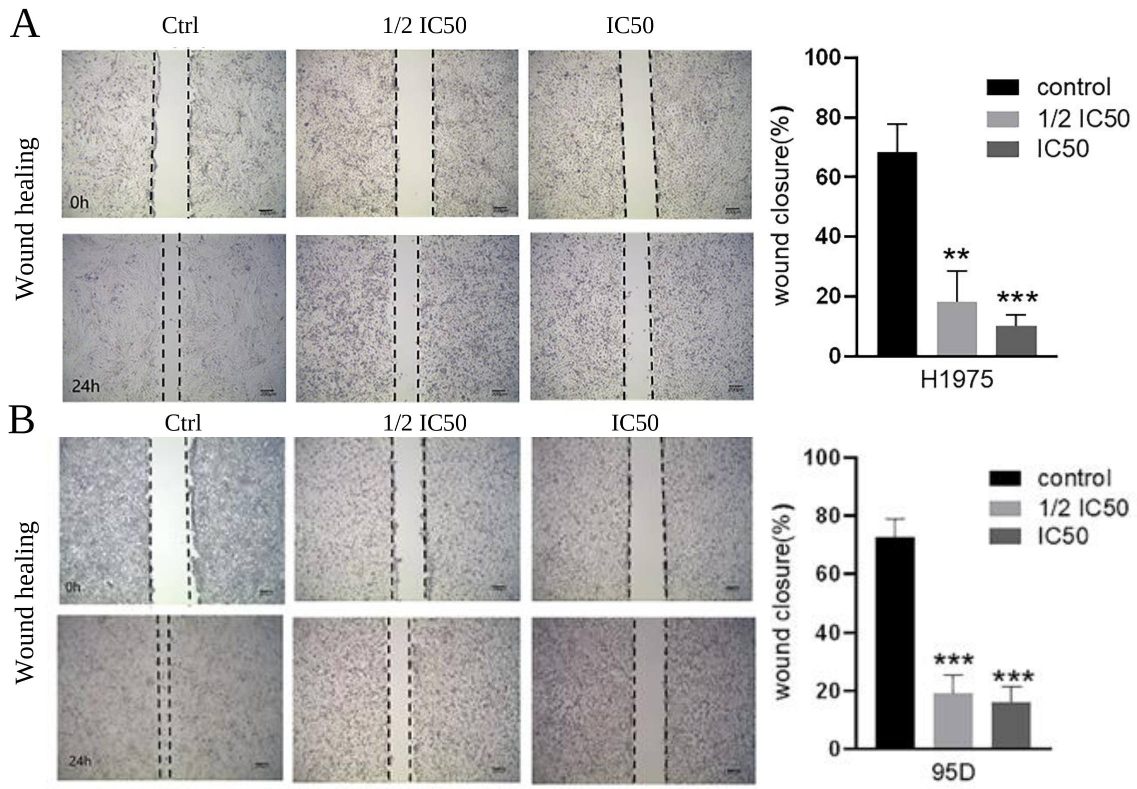

Supplementary Figure 1. FYN suppresses cellular migration of lung cancer cells. FYN-treated H1975 and 95D cells were used for wound healing assay (10 × field). Data were presented as the means ± SD from three independent experiments. *, ** and *** indicate significant difference compared to the control group at $P < 0.05$, $P < 0.01$ and $P < 0.001$, respectively. A: H1975 B: 95D.

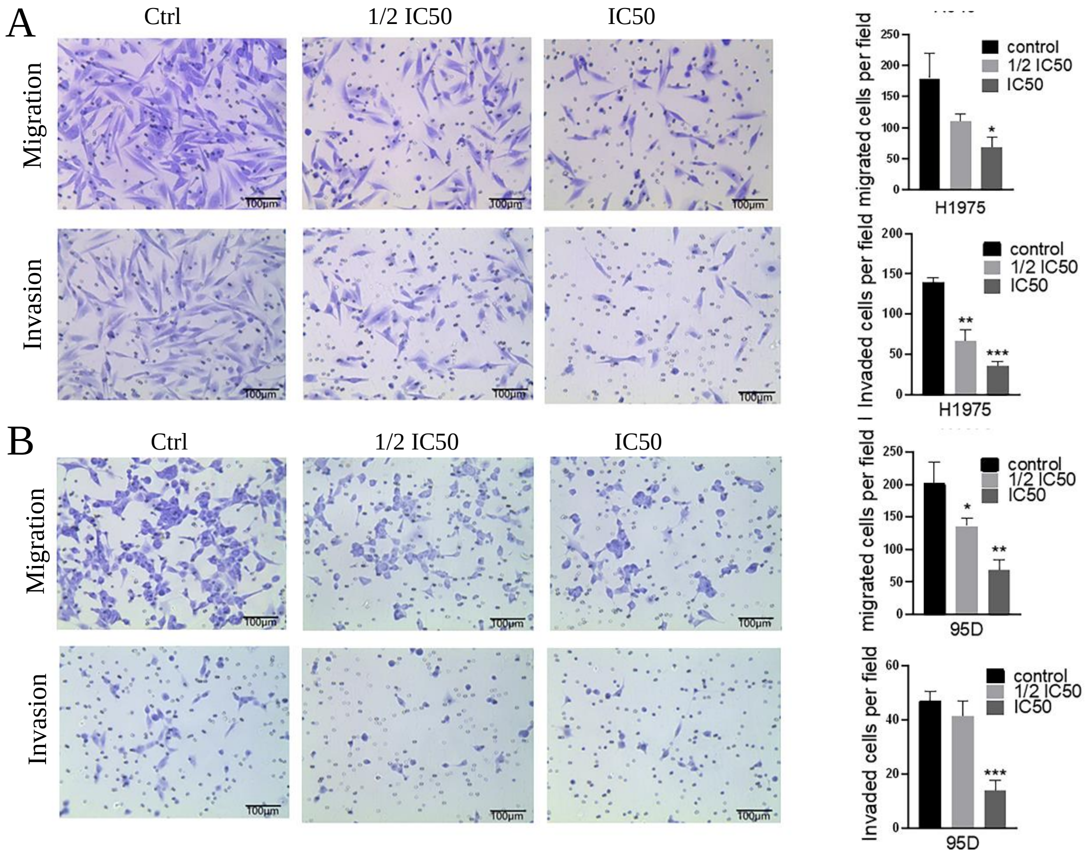

Supplementary Figure 2. FYN suppresses cellular invasion of lung cancer cells. FYN-treated A549, H1975 and 95D were used for invasion assay. Data were presented as the means ± SD from three independent experiments. *, **and *** indicate significant difference compared to the control group at $P < 0.05$, $P < 0.01$ and $P < 0.001$, respectively. A: H1975 B: 95D.

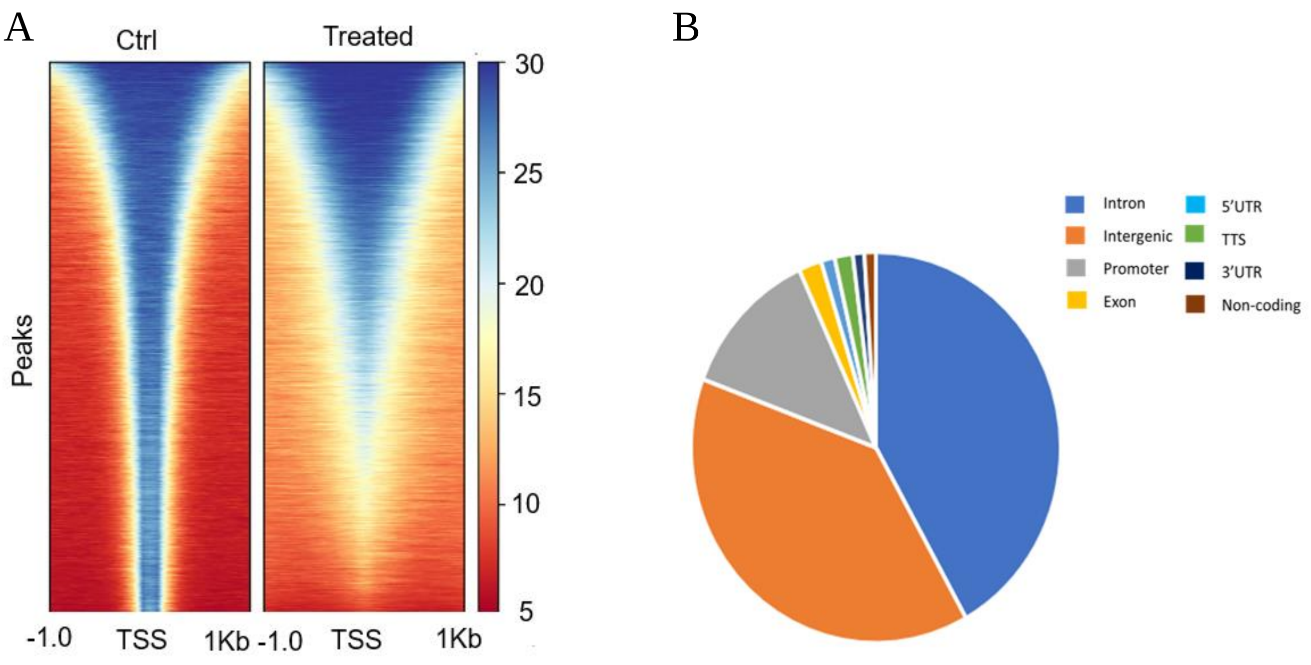

Supplementary Figure 3**.** Characterization of genome-wide chromatin accessibility in FYN-treated A549 cells. A: Heatmap indicating the average ATAC-Seq signal centered on the TSSs of the nearest genes. B: The pie chart shows the distribution of genomic regions for differiential chromatin accessibility.

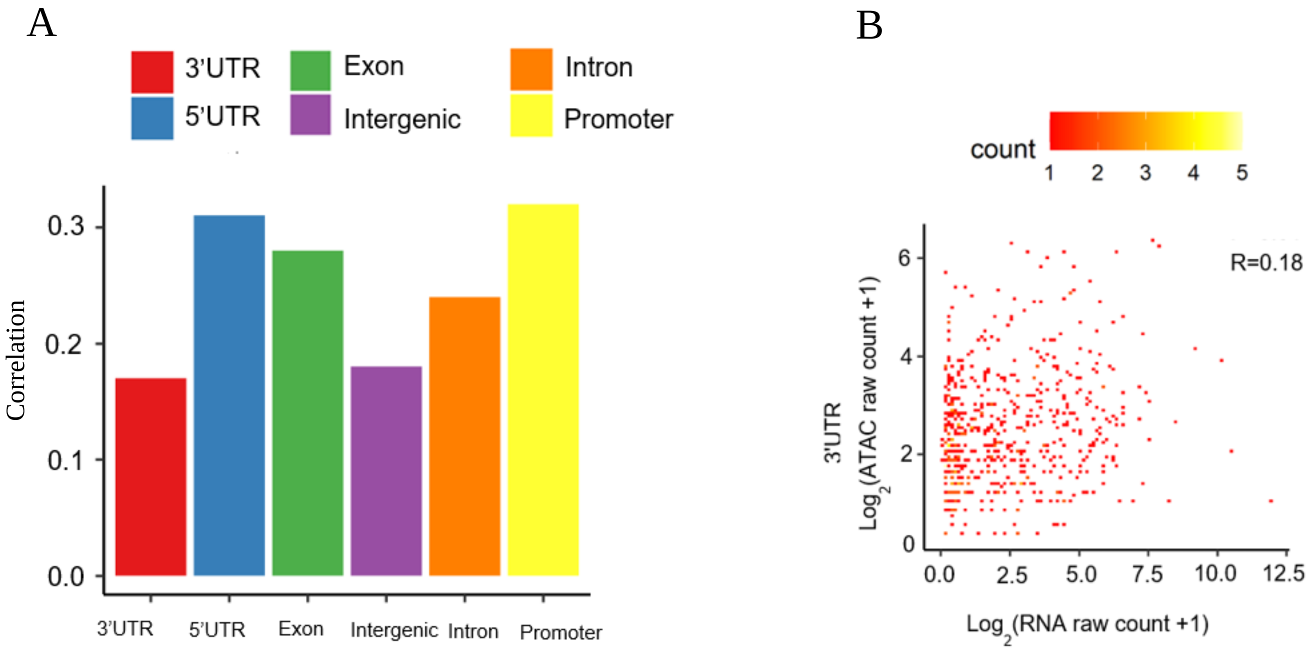

C

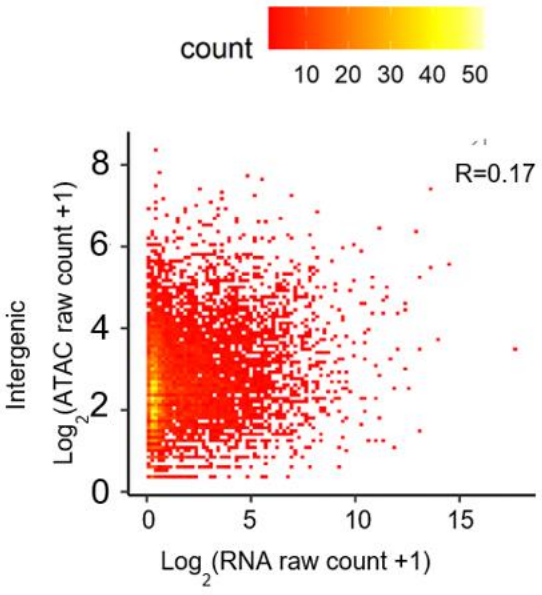

Supplementary Figure 4. A: Column of the correlation between transcript levels and total ATAC-seq signal in each genomic region. B and C: Point density plots showing the correlation between RNA-seq expression levels and ATAC-seq total tags at the intergenic and 3'UTR regions in FYN treated A549.
