# Supplementary material for: Integrative RNA-Seq and ATAC-Seq Analysis Reveals the Migration-Associated Genes Involved in Antitumor Effects of Herbal Medicine Feiyanning on Lung Cancer Cells
Source: Front Genet. 2021 Dec 21;12:799099. doi: 10.3389/fgene.2021.799099 (PMC8724546; doi:10.3389/fgene.2021.799099)
Supplement: Supplementary file 4 [file Table5.DOCX]

Raw data of wound healing assay in figure 2 and supplementary figure 1 for initial validation

A549-Wound healing：

OH：https://www.jianguoyun.com/p/DY6Yyc0Q7uP5CRjAwpUE

24H： https://www.jianguoyun.com/p/DThFmZEQ7uP5CRjjwpUE

95D-Wound healing：

0h：https://www.jianguoyun.com/p/Df1VhSkQ7uP5CRjpwpUE

24h：https://www.jianguoyun.com/p/DbCoAIIQ7uP5CRjrwpUE

H1975-Wound healing：

0h：https://www.jianguoyun.com/p/DXiOUH8Q7uP5CRjtwpUE

24h：https://www.jianguoyun.com/p/DUdCcCAQ7uP5CRjwwpUE

Raw data of transwell assay in figure 2 and supplementary figure 2 for initial validation

A549-Transwell:

Migration-24h：https://www.jianguoyun.com/p/DZj9_cwQ8pT6CRj9ipcE

Invasion-24h：https://www.jianguoyun.com/p/Dcnst-gQ8pT6CRj8ipcE

95D-Transwell:

Migration-24h：https://www.jianguoyun.com/p/DWCRRcUQ8pT6CRiBi5cE

Invasion-24h：https://www.jianguoyun.com/p/DT8McwsQ8pT6CRiCi5cE

H1975-Transwell:

Migration-24h：https://www.jianguoyun.com/p/DVHMTn8Q8pT6CRiDi5cE

Invasion-24h：https://www.jianguoyun.com/p/DZKhgwAQ8pT6CRiEi5cE
